# An Uncommon Presentation of Adrenal Cyst with Subclinical Cushing's Syndrome: A Diagnosis Dilemma

**DOI:** 10.1155/2021/6662492

**Published:** 2021-08-31

**Authors:** A. Tahri, W. Abdellaoui, S. Benyakhlef, K. Boujtat, I. Mahroug, I. Kamaoui, A. Barki, F. Kettani, S. Rouf, H. Latrech

**Affiliations:** ^1^Department of Endocrinology-Diabetology, Mohammed VIth University Hospital, Faculty of Medicine and Pharmacy of Oujda, Mohammed the First University, Oujda, Morocco; ^2^Department of Urology, Mohammed VIth University Hospital, Faculty of Medicine and Pharmacy of Oujda, Mohammed the First University, Oujda, Morocco; ^3^Department of Radiology, Mohammed VIth University Hospital, Faculty of Medicine and Pharmacy of Oujda, Mohammed the First University, Oujda, Morocco; ^4^United Nations Center of Cytopathology, Rabat, Morocco; ^5^Laboratory of Epidemiology, Clinical Research and Public Health, Faculty of Medicine and Pharmacy of Oujda, Mohammed the First University, Oujda, Morocco

## Abstract

Adrenal cysts are a rare entity that is usually nonfunctional and asymptomatic. Their association with adrenal neoplasms was rarely described in the literature. We report a unique case of a 40 -year-old woman who was referred for evaluation of a left adrenal incidentaloma with subclinical Cushing's syndrome. The tumor was suspicious for malignancy regarding computed tomography scan (CT scan) features. Therefore, a laparoscopic left partial adrenalectomy was performed. Pathology examination showed multilocular spaces lined by endothelial cells which are compatible with endothelial adrenal cyst, associated to an adrenocortical adenoma. We further discuss the management of adrenal cyst with review of the literature.

## 1. Introduction

Adrenal cysts are rare cystic masses that arise from the adrenal gland. They are usually nonfunctional and asymptomatic measuring less than 10 cm. These lesions may occur simultaneously with other adrenal tumors associated with hormonal hypersecretion. Only few papers describe the association between an adrenal cyst and adrenocortical adenoma.

We report one of the rare cases of a functioning adrenal cyst incidentally discovered.

## 2. Case Presentation

A 40-year-old woman was referred to our endocrinology department to evaluate a left adrenal incidentaloma discovered when a CT scan was performed because of her history of ureteral stones revealing a left adrenal mass of 34 × 27 mm. She had also hypertension treated with amlodipine 10 mg 2 months ago and did not complain neither of headache nor diaphoresis or palpitations.

No clinical features of Cushing's syndrome were noticed on physical examination regardless of moderate obesity with android distribution (weight: 94 kg, body mass index 30 kg/m^2^, and waist size 105 cm). But, her blood pressure levels were not well controlled under amlodipine 10 mg.

Laboratory examination showed a normal value of 24-hour urine metanephrines/normetanephrine, plasma renin, and plasma aldosterone concentration (Table 1).

Her plasma levels of midnight cortisol of two different days were 5, 2–4, and 2 ug/dL (normal value: 1.8 ug/dL with the immunoassay technique), urine free cortisol values of three different days were 36.3, 42.8, and 3.25 ug/day (normal value: 4.3–176), and serum dehydroepiandrosterone sulfate level was 119 ug/dL (normal value: < 600 ug/dL with the chemiluminescence method). However, her plasma adrenocorticotropic hormone (ACTH) concentration was at the low limit: 10.4 ng/L (normal value: 10.3–48.2 with the electrochemiluminescence method), and the overnight 1 mg dexamethasone suppression test (2.7 *μ*g/dL; reference range <1.8 *μ*g/dL) besides the low-dose dexamethasone suppression test (DST) showed an absence of cortisol suppression, which was consistent with the European Society of Endocrinology criteria of autonomous cortisol secretion [1].

Laboratory tests concerning associated comorbidities showed an increased level of total cholesterol: 2.23 g/L (normal value < 2 g/L) and triglyceride: 3.21 g/l (normal value < 1,5 g/L) and decreased level of HDL cholesterol: 0.47 g/L (normal value: < 0.60 g/L). Fasting blood sugar level was 0.85 g/L (N: 0.70–1.10 g/L) and hemoglobin A1c level (HbA1c) was 5.2%. The 75 g oral glucose tolerance test noted an impaired glucose tolerance. Bone density was normal.

**Figure 1:**
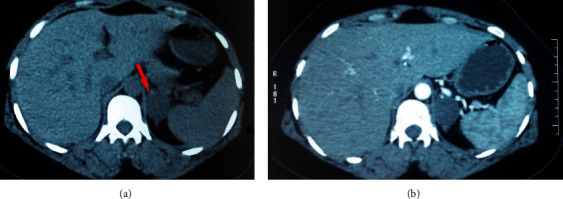
Unenhanced computed tomography scan (a) of the abdomen showing a unilocular left adrenal cyst, without wall's enhancement after contrast injection (b).

The CT scan revealed a rounded well-defined hypodense lesion of the left adrenal. Its size increased to 44 × 29 mm. Spontaneous density was 22 Hounsfield unit (HU) (Figure 1(a)), and absolute washout reached 50%. The wall of this lesion did not enhance after contrast injection, and calcification was detected (Figure 1(b)). The right adrenal gland was normal.

According to the clinical, laboratory, and imaging findings, the left adrenal mass was suspicious. Laparoscopic retroperitoneal cystic resection and left partial adrenalectomy were performed. It is the elective surgical procedure adopted by our urologist, for numerous advantages, especially for a shorter surgery duration and a minor intraoperative bleeding. Hydrocortisone replacement was not administered after the surgery because the patient did not have signs or symptoms of adrenal insufficiency.

At three months after surgery, her weight turned to 88 kg and her hypertension was stabilized under amlodipine 10 mg. Plasma 8 am ACTH concentration increased to 28 pg/mL, and plasma cortisol concentration decreased compared with her preoperative levels (Table 2). Moreover, plasma cortisol concentration was suppressed in reaction to the overnight 1 mg DST without impaired glucose tolerance (Table 2).

Pathological analysis of the resected tumor reported a 4 ^*∗*^ 2.3 ^*∗*^ 1.7 cm mass weighing 13 g with a partially yellowish cystic appearance.

**Figure 2:**
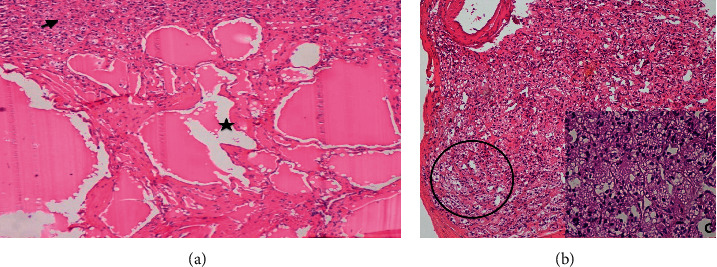
Histological study of the lesion: (a) a cystic space lined by flattened endothelial cells filled with blood (star) associated to a proliferation of well-defined clear cells (arrow) (H&Ex100). (b) Alveolar disposition of the clear cells (circle) (H&Ex200). (c) Adrenocortical adenoma (H&Ex400).

Histological examination of the different specimens showed a cystic cavity with a fibrous wall and constituted of multilocular spaces lined by endothelial cells and filled with red blood cells (Figure 2(a) (star)). This morphological feature was consistent with an endothelial cyst. A proliferation of well-defined clear cells was found at the rim of this lesion (Figure 2(a) (arrow)) arranged in an alveolar pattern (Figure 2(b)) without cytonuclear atypia (Figure 2(c)). According to Weiss's criteria, these pathological features were suggestive of an adrenocortical adenoma. This finding suggested the diagnosis of adrenocortical adenoma [2].

## 3. Discussion

Adrenal cysts are generally rare, and around 600 cases have been documented in the literature so far [3, 4]. The incidence of adrenal cysts in autopsy series ranges from 0.06% to 0.18%. They may occur at any age, although most of them may appear in the 3rd to 4th decade of life with a female preponderance [4, 5]. Our case was in the same range of age. Cyst size varies from millimeters to exceptional giant lesions above 20 cm. They may be unilocular or multilocular, usually solitary, involving the right and left adrenal glands with equal frequency. Neri and Nance [4] reported an incidence of 7% malignant forms, classically within the pseudocyst subclassification of adrenal cysts.

Clinical symptoms are related to the position and size of these benign cysts, although abdominal pain, gastrointestinal disorders, dyspnea, and palpable mass can be noted in large cysts. Besides, intracystic hemorrhage, infection, and hypertension have been also reported [6]. Our patient was asymptomatic with poorly controlled hypertension suggesting hormone secretion.

In 1966, Foster [7] classified 115 adrenal cysts into 4 histopathological types: epithelial cysts (9%), parasitic cysts (7%), particularly echinococcal type, pseudocysts (39%), and endothelial cysts (45%). Data from this evaluation demonstrated that endothelial cysts were the most common subtype. Nevertheless, Neri and Nance [4] considered pseudocyst as the most common subtype regarding their review of 515 from a total of 613 adrenal cysts with available pathology reports.

Endothelial cysts, also known as “simple cysts,” are usually thin-walled and multilocular measuring less than 2 cm. They are defined by their smooth and flattened endothelial lining, in addition to their yellow-tinged serous fluid [7]. They can be subcategorized histopathologically into angiomatous cysts and lymphangiomatous cysts which are the most common subtypes. Diagnosis can be established using the hematoxylin and eosin staining, although the immunohistochemical examination is essential to distinguish the lymphangiomatous subtype using podoplanin (D2-40) which constitutes a marker of lymphatic endothelium, from the angiomatous subtype using the angiomatous marker (CD31 and CD34) [5]. Pathological examination of our resected tumor using hematoxylin and eosin staining lead to the diagnosis of an endothelial cyst based on the presence of a cystic cavity surrounded by a fibrous wall and composed of multilocular spaces lined by endothelial cells and filled with blood.

Endothelial cysts are typically described at CT imaging as unilateral, with different sizes, thin walls (<3.5 mm), smooth borders, and multilocular structure besides septal calcifications [8]. They have a low attenuation coefficient usually less than 20 Hounsfield units (HU) and do not enhance after contrast media injection [9]. The content of these endothelial cysts is more hemorrhagic than any other subtype which may increase the attenuation coefficient [10]. The CT scan in our case exhibits typical features of an endothelial cyst, but a malignancy cannot be neglected as the lesion's size exceeded 4 cm knowing that the spontaneous density was high at 22 HU and the absolute washout was below 50% [1].

Since functioning adrenal cysts have been described in the literature [9], all patients with this tumor (especially in the presence of arterial hypertension) should undergo adrenal functionality investigations [11, 12]. Measurements of 24-h urinary metanephrines are the most used tests for pheochromocytoma identification [1, 11, 12] and the ratio between morning plasma aldosterone and plasma renin activity (PRA) is mandatory in hypertensive patients, in order to identify aldosterone-producing tumors [1, 11, 12]. Evaluation of 24-h urinary cortisol and the overnight 1 mg dexamethasone suppression test are the most used diagnostic methods [1, 11] for the identification of hypercortisolism. Otherwise, morphological imaging (CT scan or MRI) or functional studies (MIBG scan for suspected pheochromocytoma) help with determining size, morphological features (cystic contents, density, wall thickness, and contrast enhancement), and relationship with adjacent structures, since malignancy is reported [10, 11]. Nevertheless, calcifications are not pathognomonic of malignant lesions [13].

Only a few case series and reports of endothelial cysts have been documented in the literature, and their association with other adrenal neoplasms is even uncommon. In fact, Erickson et al. reported only one case of endothelial cyst associated with pheochromocytoma [14], and Nigawara et al. were the first who described a case of adrenocortical adenoma associated with an endothelial cyst [15]. Yamada et al. also reported a case associating an endothelial cyst, an adrenocortical adenoma, and a myelolipoma. [16], and more recently, this rare entity has been delineated by Hong et al. [17]. To the best of our knowledge, this is the fourth reported case of adrenocortical adenoma associated with an endothelial cyst.  Table 3 shows the three previously reported cases of endothelial cysts associated to an adrenocortical adenoma with the present case.

Our case supported what was previously described, and the gender was the same in the three cases except for the third one. The masses were incidentally discovered in the CT scan. Clinically, our patient had poorly controlled hypertension with obesity just as the case reported by Nigawara et al. [15]. Biological assessment of our case revealed an autonomous secretion of cortisol with a disturbed lipid profile and an impaired glucose tolerance, and the same findings were found in the case of Nigawara et al. The CT scan of the first case was highly suggestive of a left adrenocortical carcinoma because of the large size and the heterogeneity of the tumor (9 × 7 cm), while the second case a left adrenal myelolipoma was suspected. The CT scan of our patient was pathognomonic of an adrenal cyst, but the presence of a high spontaneous density with absolute washout below 50% was responsible of a diagnostic dilemma. On microscopic examination, the proliferation of clear cortical cells without nuclear atypia or mitotic activity was compatible with an adrenocortical adenoma, which was found in all the cases. Moreover, immunohistochemical study analysis of steroidogenic enzymes provides important information to asses the hyperfunctioning pattern of the adrenocortical adenoma [15, 16]. This includes the search for expression of the following enzymes: P450 side-chain cleavage (P450scc), 3*β*-hydroxysteroid dehydrogenase (3*β*HSD), P450c17 *α*-hydroxylase (P450c17) and P450 21 hydroxylase (P450c21)1, and DHEA-sulfotransferase (DHEA-ST). This exploration was carried out in case 1 and case 2; however, it was not made in our case because of economical issues.

The pathogenesis of an adrenal endothelial cyst formation within an adrenocortical adenoma is still a mysterious issue. Several theories suggested that the cyst may arise from a preexisting vascular hamartoma [5, 14] or lymphangiectasis [14] or intraparenchymal hemorrhage. In the present case, no apparent abnormal vascular channels have been identified.

Up to now, no guidelines have been established on the management and treatment of cystic adrenal lesions because of the low incidence of these lesions and the difficulty in achieving a definitive preoperative diagnosis. These particular cases require a multidisciplinary team when malignancy is suspected, a hormone secretion excess has been established, and when surgery is envisaged [1]. Indications for adrenal cystic lesions' surgery include a size exceeding 5 cm because of the risk of hemorrhage or other secondary complications, the presence of symptoms, endocrine abnormalities, and suspicion of malignancy [18–20]. All other asymptomatic, small lesions can be only followed by imaging, although no screening protocol has been described [20]. Fine-needle aspiration biopsy cytology of the cystic lesion has been suggested in case if the mass is hormonally inactive specifically excluding pheocromocytoma with nonbenign imaging in which pathology results would directly change management [1, 21]. In 1992, Assalia and Gagner [22] performed the first laparoscopic adrenalectomy which is considered as the gold standard in the treatment of adrenal tumors [11, 18].

Two procedures of laparoscopic adrenalectomy have been described in the literature. The transperitoneal approach is more appropriate to resect large tumors because it allows evaluation of the peritoneal space. However, the retroperitoneal technique provides good visualization of the retroperitonal anatomy, direct access to the adrenal tumor, and short duration of surgery and minimizes operative bleeding. Several studies have shown that the outcomes of the retroperitonal approach were superior to those of transperitoneal approches regarding adrenal tumors resection. Nevertheless, there is a lack of evidence regarding the effectiveness of the two approaches' comparison in patients [23].

Different methods have been suggested, and complete adrenalectomy was recommended by most authors [11, 24]. According to other papers, adrenal-sparing surgery may be performed after excluding hormone secretion, when the risk of malignancy is considered to be low or when the cyst is found to be large [18]. Furthermore, other centers perform adrenal-sparing resections even in the setting of functioning active tumors such as pheochromocytomas [25]. Moreover, during the last years, partial adrenalectomy has become an accepted alternative in the treatment of adrenal tumors [25, 26], especially for small ones [25]. He et al. [27] performed laparoscopic partial adrenalectomy in 87 Cushing's adenomas and showed that this procedure had extremely low morbidity and achieved an excellent outcome.

In our case, a small cystic tumor discovered at the time of the surgical exploration allowed for partial adrenalectomy.

Open surgery should be advocated either in complicated cases, larger cyst size, or preoperative evidence of massive compression or infiltration of adjacent structures to avoid mini-invasive approaches' complication [19].

To sum up, this is a case of adrenal cyst presenting with subclinical Cushing's syndrome. Preoperative diagnosis was made by CT scan, and laparoscopic partial adrenalectomy was performed without complications. The pathology was consistent with an adrenal endothelial cyst associated with adrenocortical adenoma. As far as we know, this is the fourth reported case of adrenal endothelial cyst associated with adrenocortical adenoma. Although it was regarded as a benign tumor in our particular case, the potential risk of malignancy when managing adrenal cyst should be taken into consideration before any surgery.

## Figures and Tables

**Table 1:** Value of U-metanephrine, U-normetanephrine, the plasma renin, and the plasma aldosterone concentration.

U-metanephrine (mg/day), *N*: 0.04–0.20 with the liquid chromatography-mass spectrometry technique		0.07
U-normetanephrine (mg/day), *N*: 0.07–0.38 with the liquid chromatography-mass spectrometry technique		0.14
Plasma aldosterone pg/ml radioimmunoassay method	Seated position *N*: 30–146	128
	After 1 hour of upright position *N*: 75–361	345

Plasma renin mUI/ml chemiluminescence method	Seated position *N*: 2.8–39.9	6.1
	After 1 hour of upright position *N*: 4.4–46.1	20.1

Aldosterone-renin ratio (ARR)	Seated position *N*: < 64	58
	After 1 hour of upright position *N*: <64	48

**Table 2:** Comparative table of laboratory data before and after surgery.

Variable	Before surgery	3 months after surgery
Midnight cortisol^*∗*^ (ug/dL) Normal: < 1.8	5.2–4.2	1–1.7
u-free cortisol^*∗∗*^ (*μ*g/day) normal: 4.3–176	36-42-3.25	23-13-9
Plasma ACTH (ng/L) 8 AM Normal: 10.30–48.30	10.7	28.5
Cortisol plasma after 1 mg DST (ug/dL) Normal <1.8	2.7	0.7
Plasma glucose at 120 min in 75 g OGTT (mg/dL) Normal <1.40	1.78	1.10

ACTH: adrenocorticotropic hormone; DST: overnight dexamethasone suppression test; DHEA-S: dehydroepiandrosterone sulfate; OGTT: oral glucose tolerance test. ^*∗*^In plasma examination, values of two different days are shown to view day-to-day variation. ^*∗∗*^In urine examination, values of three different days are shown to view day-to-day variation.

**Table 3:** Comparative table of the previous cases and the present case.

	Clinical presentation	Laboratory assessment	CT scan	Pathology findings	Treatment
Case 1 Nigawara et al., 2009 [15]	Incidental left adrenal mass in a 68-year-old Japanese woman, with obesity and hypertension	Subclinical Cushing's syndrome	Left adrenal tumor (9 × 7 cm) with highly heterogeneous configuration, including foci of calcification. Contrast media stained only the rim of the tumor	Vascular cyst with hyaline degeneration (lining cells were CD34 positive) within an adrenocortical adenoma. Immunohistochemical analysis of steroidogenic enzymes showed the expression of P450scc, 3*β*HSD, P450c17, and P450c21	Laparoscopic left adrenalectomy

Case 2 Yamada et al., 2011 [16]	Incidental right renal and left adrenal tumors discovered in a 72-year-old Japanese female, with type 2 diabetes mellitus	Normal (nonfunctioning)	Right kidney: an enhanced mass, measuring 3 cm × 3 cm. Left adrenal: a well-demarcated nodule measuring 2 cm, consisting of nonenhanced and low-density areas	Right kidney: eenal clear cell carcinoma. Left adrenal: association of myelolipoma, endothelial cyst, and adrenocortical adenoma. Immunohistochemical analysis: expression of cytochrome P450c17, 3H*β*SD, and DHEA-sulfotransferase	Right nephrectomy and left adrenalectomy

Case 3 Hong et al., 2020 [17]	Incidental right adrenal mass in a 53-year-old Chinese man with type 2 diabetes	Nonfunctioning adrenal mass	A right circular low-density mass measuring (6 × 7 cm), with a multiroom separation inside the mass. The CT value is 17 HU with no significant enhancement on contrast-enhanced CT	Cystic mass composed of fibrous wall tissues with local calcification. Another mass with a fibrous capsule outside the cystic wall composed of bright and dark cells, which are arranged in acinar and flaky shapes, and a large, deformed nucleus was present in the foci. Immunohistochemistry shows that the cells stained positive for CD34, D2-40, desmin, and SMA	Laparoscopic right adrenalectomy

Case 4 The present case	Incidental left adrenal lesion discovered in a 40-year-old woman, with hypertension and obesity	Subclinical Cushing's syndrome	A rounded well-defined nonenhancing hypodense lesion of the left adrenal measuring 44 × 29 mm in size, with high spontaneous density, and the absolute washout reached 50% with calcification in the wall which did not enhance after contrast injection	Endothelial cyst within an adrenocortical adenoma	Laparoscopic cystic resection and left partial adrenalectomy
